# Thymocytes in *Lyve1-CRE/S1pr1*^f/f^ Mice Accumulate in the Thymus due to Cell-Intrinsic Loss of Sphingosine-1-Phosphate Receptor Expression

**DOI:** 10.3389/fimmu.2016.00489

**Published:** 2016-11-08

**Authors:** Akira Takeda, Mohammad Shahadat Hossain, Pia Rantakari, Szandor Simmons, Naoko Sasaki, Marko Salmi, Sirpa Jalkanen, Masayuki Miyasaka

**Affiliations:** ^1^MediCity Research Laboratory, University of Turku, Turku, Finland; ^2^Department of Immunology and Cell Biology, Graduate School of Medicine and Frontier Biosciences, Osaka University, Suita, Japan; ^3^WPI Immunology Frontier Research Center, Osaka University, Suita, Japan; ^4^Department of Microbiology and Immunology, Graduate School of Medicine, Osaka University, Suita, Japan; ^5^Department of Medical Microbiology and Immunology, University of Turku, Turku, Finland; ^6^Interdisciplinary Program for Biomedical Sciences, Institute for Academic Initiatives, Osaka University, Suita, Japan

**Keywords:** sphingosine-1-phosphate, S1PR1, thymus, Lyve1, egress

## Abstract

T cell emigration from the thymus is essential for immunological homeostasis. While stromal cell-produced sphingosine-1-phosphate (S1P) has been shown to promote thymocyte egress *via* the S1P receptor, S1PR1, the significance of S1P/S1PR1 signaling in the thymic stromal cells that surround T cells remains unclear. To address this issue, we developed conditional knockout mice (*Lyve1-CRE/S1pr1*^f/f^ mice) in which *S1pr1* was selectively targeted in cells expressing the lymphatic endothelial cell marker, Lyve1. In these mice, T cells were significantly reduced in secondary lymphoid tissues, and CD62L^+^ mature CD4 and CD8 single-positive (SP) T cells accumulated in the medulla failed to undergo thymus egress. Using a *Lyve1* reporter strain in which Lyve1 lineage cells expressed tdTomato fluorescent protein, we unexpectedly found that a considerable proportion of the thymocytes were fluorescently labeled, indicating that they belonged to the Lyve1 lineage. The CD4 and CD8 SP thymocytes in *Lyve1-CRE/S1pr1*^f/f^ mice exhibited an egress-competent phenotype (HSA^low^, CD62L^high^, and Qa-2^high^), but were CD69^high^ and lacked S1PR1 expression. In addition, CD4 SP thymocytes from these mice were unable to migrate to the periphery after their intrathymic injection into wild-type (WT) mice. In contrast, WT T cells could migrate to the periphery in both WT and *Lyve1-CRE/S1pr1*^f/f^ thymuses. These results demonstrated that thymocyte egress is mediated by T cell-expressed, but not stromal cell-expressed, S1PR1 and caution against using the *Lyve1-CRE* system for selectively gene deletion in lymphatic endothelial cells.

## Introduction

Sphingosine-1-phosphate (S1P) is a polar lipid mediator that is intracellularly generated from sphingomyelin by the successive actions of ceramidase and the sphingosine kinases, such as Sphk1 and Sphk2, and is transported out of the cells by S1P transporters, such as Spns2 (1). Because the S1P concentrations are much higher in blood and lymph than in the parenchyma of lymphoid tissue, S1P gradients are thought to form between lymph and lymphoid tissues and to mediate the lymphocyte egress from lymphoid tissues *via* the S1P receptor S1PR1, expressed on lymphocytes (2, 3).

The S1PR1 is encoded by the *S1pr1* gene in mouse and is a G protein-coupled receptor (GPCR) originally identified by its involvement in endothelial cell (4). S1PR1 couples mainly to G_i/o_ proteins to induce activation of the Ras–ERK, PI3K–Akt, and small GTPases (Rac and Rho) signaling pathways (5). Both *S1pr1*-deficient mice (6) and *Tie2-CRE/S1pr1*^f/f^ mice (7), in which *S1pr1* is selectively disrupted in endothelial cells, die during embryogenesis due to vascular network abnormalities. S1PR1 is also highly expressed in lymphocytes, and as described above, lymphocyte-intrinsic S1PR1 is thought to regulate lymphocyte egress from the thymus (8–10) as well as from secondary lymphoid tissues (9).

Paradoxically, however, S1PR1 activation *in vivo* is found to occur predominantly in the CD31-expressing vascular structures, and not in the majority of lymphocytes in lymphoid tissues, including the thymus, under homeostatic conditions (11). Given that thymocytes leave the thymus *via* blood vessels (10, 12) and also lymphatics (12–14), the finding that S1PR1 is activated in the thymic vascular endothelial cells suggests that the thymic vasculature (blood vessels and lymphatics) may also play a role in mediating thymocyte egress to the periphery.

The lymphatic vessel endothelial hyaluronan receptor 1 (Lyve1) is a type I integral membrane protein bearing a Link module that binds hyaluronan, one of the most abundant glycosaminoglycans in the extracellular matrix (15). Lyve1 has been shown to bind and internalize hyaluronan (16), and hyaluronan binding activates intracellular signaling that promotes lymphatic endothelial cell proliferation (17). Since *Lyve1*-null mice exhibit normal lymphatic development and function (18), Lyve1 loss may be compensated by other hyaluronan receptors, or the function of Lyve1 may be more specific than previously thought. Because Lyve1 is expressed by most lymphatic endothelial cells, it has been widely used as a lymphatic endothelial-specific marker, although its expression has been reported in murine ocular (19) and adipose (20) tissue macrophages, murine embryonic blood vessel endothelial cells (21), and in human hepatic blood vessel endothelial cells (22). More recently, Lee demonstrated that Lyve1 is expressed in a fraction of hematopoietic stem cells and in blood vessel endothelial cells in the mouse yolk sac, suggesting that this protein is expressed during hematopoiesis and vascular development in mice (23).

*Lyve1-eGFP-CRE* mice express Cre recombinase and enhanced green fluorescent protein (eGFP) under control of the *Lyve1* promoter (24). Researchers have used these mice for the conditional ablation of genes in the lymphatic endothelium by crossing them with strains carrying *loxP*-flanked genomic segments of interest (24–26). Here, we developed *Lyve1-CRE/S1pr1*^f/f^ mice to investigate the role of S1PR1 signaling in lymphatic-dependent thymocyte egress. In these mice, mature CD4 and CD8 single-positive (SP) T cells were unable to leave the thymus and accumulated in the medulla. Unexpectedly, we found that S1PR1 expression was absent in a substantial proportion of the thymocytes, suggesting that *Lyve1* may normally be expressed in T cells. Tracking the Lyve1 lineage cells by using a *Lyve1-CRE/R26-*Tdtomato reporter strain confirmed that *Lyve1* was expressed in a substantial proportion of peripheral T cells as well as in thymocytes, particularly those in the thymic medulla, which are thought to emigrate from the thymus (10, 27, 28). Intrathymic injection studies confirmed that *Lyve1-CRE/S1pr1*^f/f^ T cells were unable to leave the thymus, whereas WT T cells were capable of leaving both the WT and *Lyve1-CRE/S1pr1*^f/f^ thymuses. Thus, our findings suggest that Lyve1 lineage thymocytes migrate from the thymus in an S1PR1-dependent manner, whereas the S1PR1 signaling in thymic stromal cells appears to be dispensable for thymocyte egress. Finally, our results also indicate that caution should be used when employing the *Lyve1-CRE* system to selectively target genes in lymphatic endothelial cells.

## Materials and Methods

### Ethics Statement

All mice were housed at the Central Animal Laboratory at the University of Turku. The animal experiments were approved by the Ethical Committee for Animal Experimentation (under license number 5587/04.10.07/2014) in Finland, and they were performed according to the 3R-principle and in adherence with the Finnish Act on Animal Experimentation (497/2013).

### Mice

The B6.129P2-*Lyve1^*tm1.1(EGFP/cre)Cys*^*/J (*Lyve1-CRE*), B6.129S6(FVB)-*S1pr1^tm2.1Rlp^*/J (*S1pr1*^f/f^), B6.Cg-*Gt(ROSA)26Sor^tm14(CAG^*^−^*^tdTomato)Hze^*/J (*R26*-tdTomato), C57BL6-Tg (CAG-EGFP) 10sb/J (beta-actin-eGFP), and B6.Cg-Tg(CAG-DsRed*MST)1Nagy/J (beta-actin-DsRed) mice were purchased from Jackson Laboratory. The *Lyve1-CRE* mice were bred with *S1pr1*^f/f^ or *R26*-tdTomato mice to generate *Lyve1-CRE/S1pr1*^f/f^ mice for functional studies and to generate *Lyve1-CRE/R26*-tdTomato mice for imaging experiments.

### Immunohistochemistry

For immunohistochemical analysis of the thymus and lymph nodes (LNs), 6-μm thick frozen sections were fixed with acetone at −20°C and incubated with Alexa647-conjugated ERTR7 (Santa Cruz), Alexa488-conjugated anti-Lyve1 (223322; R&D Systems), and rabbit polyclonal anti-S1PR1 antibodies (H60; Santa Cruz), followed by incubation with Alexa546-conjugated goat anti-rabbit IgG antibody (Life Technologies). The images were captured using a confocal microscope (LSM 780; Zeiss). To detect Qa-2^+^ cells and tdTomato^+^ cells, the thymuses were fixed with 4% paraformaldehyde and then subsequently embedded in 4% low-melting agarose (Lonza) in PBS and cut into 200-μm thick sections using a vibratome (VT1200S; Leica). The sections were incubated overnight at 4°C in PBS containing 0.1% BSA and 1% Triton X-100, and Hoechst 33342 (Life Technologies), Alexa647-conjugated anti-Qa-2 antibody (695H1-9-9; BioLegend), DyLight550-conjugated anti-CD31 antibody (MEC13.3; Novus Biologicals), or APC-conjugated anti-CD31 antibody (MEC13.3; BioLegend). The images were captured using a spinning disk confocal microscope (Intelligent Imaging Innovations) and processed using Imaris software (Bitplane). Quantitation of the cortical and medullary area of the thymus was performed using ImageJ software (National Institute of Health).

### Flow Cytometry

Thymocytes and lymphocytes were obtained from the thymus, LNs, and spleen by mechanically dissociating the tissues. Peripheral blood lymphocytes were isolated using Ficoll-Paque PLUS (GE Healthcare). Stromal cells in the thymus and LNs were isolated as described previously, with minor modifications (29). Briefly, the tissues were digested with 0.8 mg/ml dispase (Gibco), 0.2 mg/ml Collagenase P (Sigma), and 0.1 mg/ml DNase (Roche). The resulting single-cell suspensions were enriched for non-hematopoietic stromal cells by using CD45 microbeads (Miltenyi Biotech).

The lymphocytes and stromal-enriched cells were resuspended in PBS containing 1 mM EDTA, 2% fetal calf serum, and 0.1% sodium azide and incubated with the following antibodies: APC-Cy7-conjugated anti-CD4 (GK1.5), PerCP-Cy5.5-conjugated anti-CD8 (53-6.7), Pacific Blue-conjugated anti-B220 (RA3-6B2), BV510-conjugated anti-CD62L (MEL-14), FITC-conjugated anti-CD44 (IM7), PE-Cy7-conjugated anti-CD69 (H1.2F3), Alexa647-conjugated anti-Qa-2 (695H1-9-9), Pacific Blue-conjugated anti-HSA (M1/69), anti-6C10 (SM6C10) (30), V450-conjugated anti-CD45 (30-F11), PE-Cy7-conjugated anti-Gp38 (8.1.1), or APC-conjugated anti-CD31 (MEC13.3), all of which were purchased from either BioLegend or BD Biosciences except anti-6C10. To detect 6C10, lymphocytes were further incubated with phycoerythrin-conjugated goat anti-rat IgM antibody (Southern Biotech).

S1PR1 expressed on thymocytes and stromal cells was detected, as described previously (31). Briefly, single-cell suspensions were incubated with rat anti-S1PR1 monoclonal antibody (40 μg/ml, 713412; R&D Systems) followed by incubation with phycoerythrin-conjugated goat anti-rat IgG antibody (Southern Biotech).

All flow cytometric data were acquired on an LSR Fortessa (BD Biosciences) and analyzed using FlowJo software (FlowJo, LLC).

### Intrathymic Injection

CD62L^+^ CD4^+^ SP cells were isolated from the thymus using a mouse CD4^+^ T cell isolation kit (StemCell Tech), followed by the positive selection of CD62^+^ cells (Miltenyi Biotech). To label the cells, they were incubated with 5 μM CFSE or 5 μM CMTMR for 30 min at 37°C in RPMI containing 2% FCS. The cells (1 × 10^6^ cells in 10 μl PBS) were then injected into the thymus of recipient mice, as described previously, with some modifications (32). Young female mice (less than 4 weeks old) were used as the recipient mice to control thymus size, and the single-cell suspension was injected directly through the skin into the thoracic cavity immediately above the sternum using a 10-μl Hamilton syringe equipped with a 33-gauge needle. Two days after the injection, the recipient mice were sacrificed, and the donor-derived cells in the thymus, blood, spleen, and LNs were quantified by flow cytometry.

### Adoptive Homing Assays

Lymphocytes were collected from LNs of beta-actin-eGFP mice. The cells (1 × 10^7^ cells in 200 μl PBS) were then intravenously injected into the recipient mice. Twelve hours after the injection, the recipient mice were sacrificed, and the donor-derived cells in the inguinal LNs were quantified by flow cytometry.

### Quantitative PCR Analysis

CD62L^+^CD4^+^ cells were isolated from the thymus, as described above, prior to RNA purification (RNeasy Plus Micro, QIAGEN). cDNA was generated using Superscript VILO cDNA synthesis kit (Life Technologies) and analyzed by a quantitative TaqMan RT-PCR method with the primers and probes designed by Universal ProbeLibarary Assay Design Center (Roche). *S1pr1* primers; forward: CGGTGTAGACCCAGAGTCCT, reverse: AGCTTTTCCTTGGCTGGAG, *Actb* primers; forward: CTAAGGCCAACCGTGAAAAG, reverse: ACCAGAGGCATACAGGGACA. The expression values were normalized using *Actb* expression as endogenous controls.

### Statistical Analysis

Differences between groups were evaluated with Student’s *t*-test for single comparisons or one-way ANOVA, followed by *post hoc* Tukey tests for multiple comparisons. The statistical analyses were performed using Prism software (GraphPad). A *P-*value <0.05 was considered to be statistically significant. Data are presented as the mean ± SD unless otherwise indicated.

## Results

### T Cells Are Substantially Reduced in the Secondary Lymphoid Tissues of the *Lyve1-CRE/S1pr1*^f/f^ Mice

**Figure 1:**
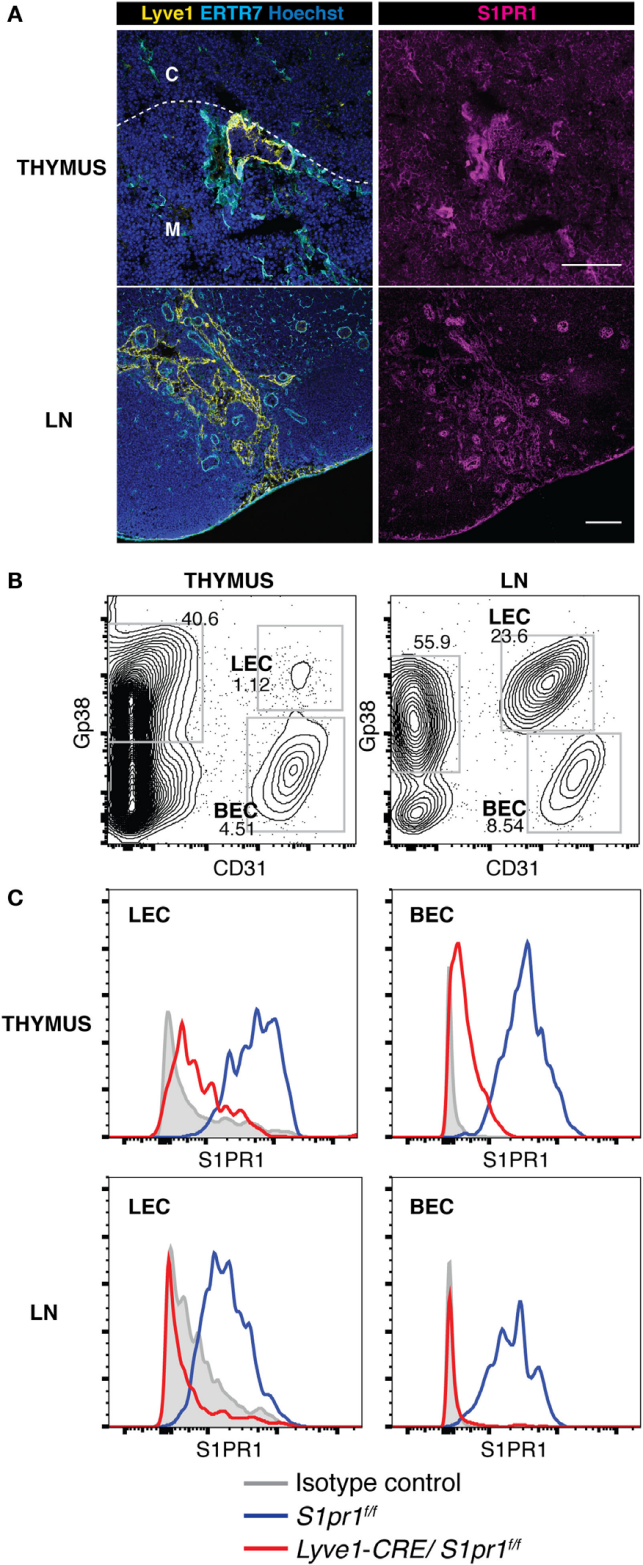
**S1PR1 is expressed in lymphatic endothelial cells of the thymus and LNs**. **(A)** S1PR1 expression was examined immunohistologically in the thymus and LNs. Lyve1-positive lymphatics were observed in the vicinity of the cortico-medullary junction (dotted line). C, cortex; M, medulla. Bars, 100 μm. **(B,C)** Flow cytometric analysis of S1PR1 expression in thymic and LN stromal cells of *S1pr1*^f/f^ and *Lyve1-CRE/S1pr1*^f/f^ mice. **(B)** CD45-negative stromal cell populations from the thymus and LNs. Lymphatic and blood vessel endothelial cells (LECs and BECs) were identified by their expression of Gp38 and CD31. **(C)** S1PR1 expression in LECs and BECs in the thymus and LNs of *S1pr1*^f/f^ (blue line) and *Lyve1-CRE/S1pr1*^f/f^ mice (red line). Data are representative of two **(A)** or three **(B,C)** independent experiments.

While T cell egress from lymphoid tissues has been shown to require S1PR1 expression on T cells (8, 9), a recent study indicates that S1PR1 activation occurs mainly in the vascular structures rather than on the T cells in lymphoid tissues (11). As shown in Figure 1, S1PR1 was readily detected immunohistologically in the vascular structures including the blood vessels and lymphatics of the thymus and LNs (Figure 1A; Figure S1 in Supplementary Material), and FACS studies confirmed the distinct S1PR1 expression in the blood endothelial cells and lymphatic endothelial cells of these tissues (Figures 1B,C). To address the functional significance of the lymphatic endothelial cell-expressed S1PR1 in lymphocyte egress from lymphoid tissues, we developed *Lyve1-CRE/S1pr1*^f/f^ mice, in which *S1pr1* was deleted selectively in Lyve1 lineage cells due to Cre-mediated excision of the loxP-flanked *S1Pr1* allele. The *S1pr1*^f/f^ mice were used as controls. As expected, *Lyve1-CRE/S1pr1*^f/f^ mice exhibited a strongly downregulated expression of S1PR1 on the lymphatic endothelial cells of the thymus and LNs (Figure 1C); however, their lymphatics were morphologically indistinguishable from those in *S1pr1*^f/f^ mice (data not shown). *Lyve1-CRE/S1pr1*^f/f^ mice also exhibited a markedly reduced S1PR1 expression on blood vessel endothelial cells (Figure 1C), suggesting that the *Lyve1* promoter is active in both lymphatic and blood vessel endothelial cells in the thymus.

**Figure 2:**
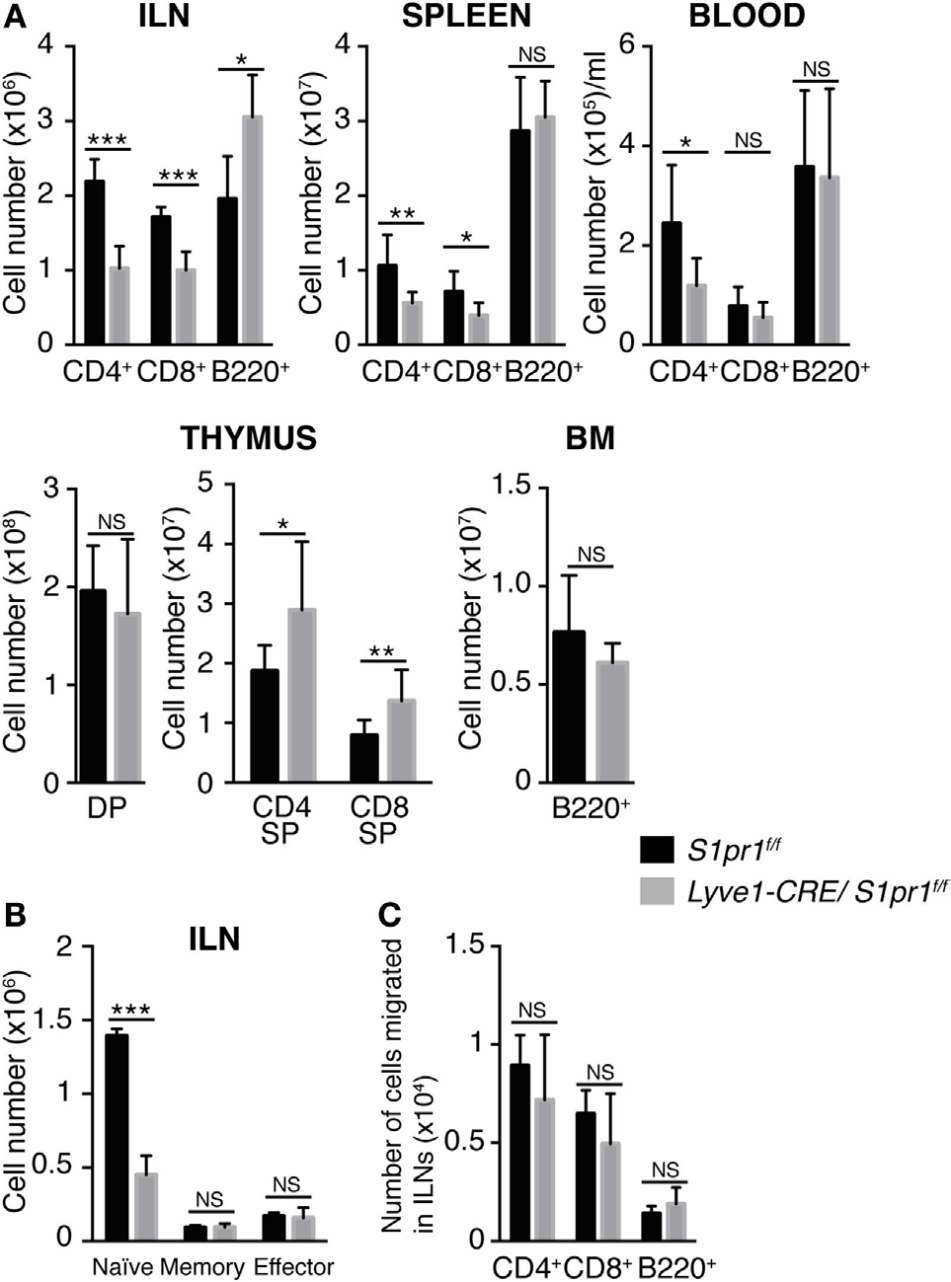
**T cells are remarkably decreased in the peripheral lymphoid tissues of *Lyve1-CRE/S1pr1*^f/f^ mice**. **(A)** T and B cell numbers in the inguinal LNs (ILNs), spleen, blood, thymus, and bone marrow (BM) of *S1pr1*^f/f^ and *Lyve1-CRE/S1pr1*^f/f^ mice. DP, CD4^+^CD8^+^ double positive cells; CD4 or CD8 SP, CD4^+^ or CD8^+^ single-positive cells. **(B)** Numbers of CD4^+^ T cell subsets in the inguinal LNs of *S1pr1*^f/f^ and *Lyve1-CRE/S1pr1*^f/f^ mice. Naïve, memory, and effector CD4^+^ T cells were defined as CD62L^+^CD44^−^, CD62L^+^CD44^+^, and CD62L^−^CD44^+^, respectively. **(C)** Migration of WT lymphocytes into the LNs of *S1pr1*^f/f^ and *Lyve1-CRE/S1pr1*^f/f^ mice. Lymphocytes from beta-actin-eGFP mice were injected intravenously into recipient mice. After 12 h, the inguinal LNs were collected, and donor-derived lymphocytes were quantified by flow cytometry. These data are pooled from three independent experiments (*n* > 4) **(A)** and are representative of two **(B,C)** independent experiments with three mice per group. These data were evaluated by Student’s *t*-test and represent the mean ± SD (*P < 0.05, **P < 0.005, and ***P < 0.0005).

Analysis of the lymphoid tissues and peripheral blood of *Lyve1-CRE/S1pr1*^f/f^ mice revealed that the number of both CD4^+^ T and CD8^+^ T cells in the LNs, spleen, and peripheral blood was substantially reduced compared with those in *S1pr1*^f/f^ control mice (Figure 2A). In contrast, there was no significant difference in the number of B cells between the two groups of mice. Further analysis showed that naïve (CD62L^high^ and CD44^low^) T cells, but not memory-type (CD62L^high^ and CD44^high^) or effector-type (CD62L^low^ and CD44^high^), T cells were markedly reduced in the LN of *Lyve1-CRE/S1pr1*^f/f^ mice compared to the control mice (Figure 2B). Analysis of the lymphocytes in the primary lymphoid organs showed that the numbers of CD4^+^ and CD8^+^ SP cells in the thymus of *Lyve1-CRE/S1pr1*^f/f^ mice were significantly increased compared to those in *S1pr1*^f/f^ mice, whereas the number of B cells in the bone marrow and CD4^+^CD8^+^ double positive (DP) cells in the thymus was comparable in the two types of mice (Figure 2A).

Because S1PR1 expression was disrupted not only in lymphatic endothelial cells but also in blood vessel endothelial cells in *Lyve1-CRE/S1pr1*^f/f^ mice (Figure 1B), we investigated whether the marked reduction in naïve T and B cells in the peripheral LNs in these mice was due to an impaired ability of the high endothelial venules to mediate lymphocyte recirculation. To this end, we performed a lymphocyte adoptive transfer assay by intravenously injecting LN lymphocytes from beta-actin-eGFP mice into either *Lyve1-CRE/S1pr1*^f/f^ mice or *S1pr1*^f/f^ mice. We then examined the migration of the eGFP^+^ cells into the recipients’ LNs. As shown in Figure 2C, adoptively transferred CD4^+^ T cells, CD8^+^ T cells, and B cells were found in comparable numbers in *Lyve1-CRE/S1pr1*^f/f^ and *S1pr1*^f/f^ LNs, indicating that *S1pr1* deletion in the Lyve1-expressing cells did not compromise the ability of high endothelial venules to mediate lymphocyte trafficking from blood to lymph. These results indicated that S1PR1 deletion in Lyve1-expressing cells reduced the number of circulating T and B cells without affecting high endothelial venule-mediated lymphocyte recirculation.

### Both CD4^+^ and CD8^+^ SP Subsets Expressing Qa-2 at High Levels Are Markedly Increased in the Thymic Medulla of the *Lyve1-CRE/S1pr1*^f/f^ Mice

**Figure 3:**
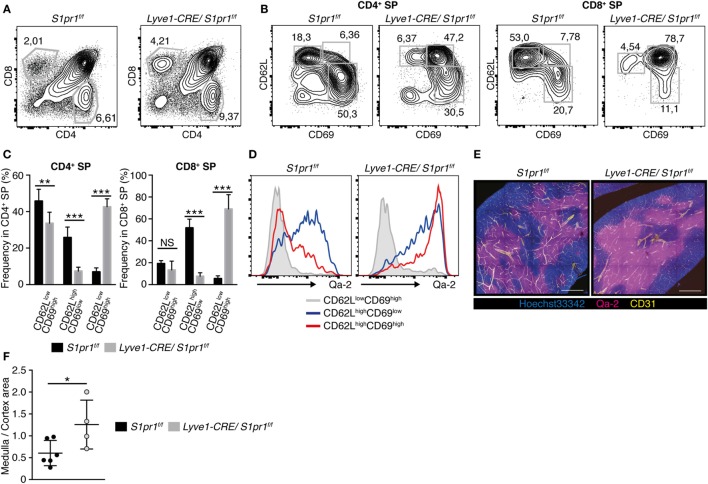
**Mature CD4^+^ and CD8^+^ SP cells are accumulated in the *Lyve1-CRE/S1pr1*^f/f^ thymus**. **(A)** Frequencies of CD4^+^ and CD8^+^ SP cells in the thymus of *Lyve1-CRE/S1pr1*^f/f^ and *S1pr1*^f/f^ mice (*n* = 5). **(B,C)** Frequencies of immature (CD62L^low^CD69^high^), mature (CD62L^high^CD69^low^), and CD62^high^CD69^high^ cells in the CD4^+^ or CD8^+^ SP subset of *S1pr1*^f/f^ and *Lyve1-CRE/S1pr1*^f/f^ mice. Data were pooled from nine mice **(C)**. The data were evaluated by Student’s *t*-test and represent the mean ± SD (**P < 0.005 and ***P < 0.0005). **(D)** Qa-2 expression in the CD62L^low^CD69^high^, CD62L^high^CD69^low^, and CD62L^high^CD69^high^ cells in the CD4^+^ SP populations of the *S1pr1*^f/f^ and *Lyve1-CRE/S1pr1*^f/f^ thymuses (*n* = 3). **(E)** Immunohistological analysis of the *S1pr1*^f/f^ and *Lyve1-CRE/S1pr1*^f/f^ thymuses. Thymus sections (200-μm thick) were stained with antibodies against Qa-2 (magenta) and CD31 (yellow), nuclear staining was performed using Hoechst 33342 (blue). Qa-2 expression was prominent in the thymic medulla. Bars, 500 μm (*n* = 2). **(F)** Quantification of the cortical and medullary area in the thymus of *Lyve1-CRE/S1pr1*^f/f^ and *S1pr1*^f/f^ mice. Sections were cut approximate middle of the thymus, and the medullary area was determined based on Qa-2 staining: *S1pr1*^f/f^ (*n* = 6) and *Lyve1-CRE/S1pr1*^f/f^ (*n* = 4). These data were evaluated by Student’s *t*-test and represent the mean ± SD (*P < 0.05).

Because naïve T cells were strongly reduced in the LNs of *Lyve1-CRE/S1pr1*^f/f^ mice, we further examined the T cell subsets in the thymus of these mice. As described above, although the *Lyve1-CRE/S1pr1*^f/f^ and *S1pr1*^f/f^ thymuses contained similar numbers of CD4^+^CD8^+^ DP cells, there were modest but reproducible increases in the proportion of CD4^+^ and CD8^+^ SP subsets in the *Lyve1-CRE/S1pr1*^f/f^ thymus (Figures 2A and 3A). Among the SP subsets, the CD62L^high^CD69^high^ cells, which barely detectable in the WT thymus, were easily detected in the *Lyve1-CRE/S1pr1*^f/f^ thymus, while the relatively immature (CD62L^low^CD69^high^) and mature (CD62L^high^CD69^low^) SP subsets were proportionately reduced in *Lyve1-CRE/S1pr1*^f/f^ compared with the *S1pr1*^f/f^ thymus (Figures 3B,C). The increased CD62L^high^CD69^high^ CD4^+^ (Figure 3D) and CD8^+^ SP subsets (data not shown) expressed high levels of Qa-2, a cell surface marker that is normally expressed strongly on CD69^low^ recent thymic emigrants (33) (Figure 3D), although CD62L^high^CD69^high^ subsets in *S1pr1*^f/f^ thymus expressed low levels of Qa-2 (Figure 3D), raising the possibility that these cells were defective in thymic egress. Consistent with this possibility, the *Lyve1-CRE/S1pr1*^f/f^ mice exhibited markedly enlarged thymic medullas (Figures 3E,F). Thus, our results indicated that S1PR1 ablation in *Lyve1*-expressing cells leads to a thymic accumulation of SP T cells, possibly due to their impaired ability to exit from the thymus.

### The Medullary SP Subsets in *Lyve1-CRE/S1pr1*^f/f^ Mice Phenotypically Resemble Egress-Competent Thymocytes in Wild-type Mice but Lack S1PR1 Expression

**Figure 4:**
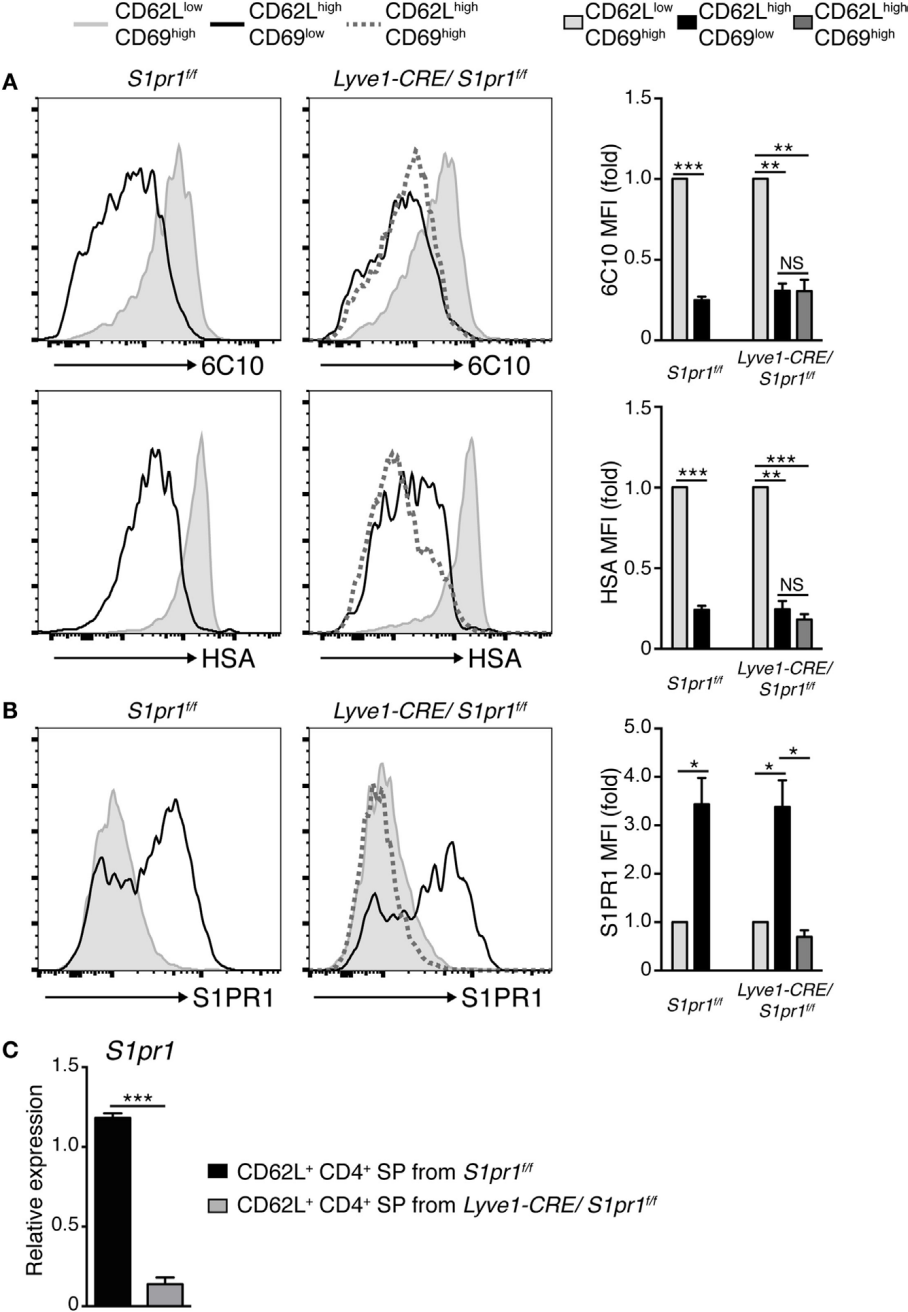
**Thymocytes accumulating in the thymus of *Lyve1-CRE/S1pr1*^f/f^ mice resemble egress-competent cells, but lack S1PR1 expression**. **(A,B)** Expression of 6C10 and heat stable antigen (HSA) **(A)** or S1PR1 **(B)** in immature (CD62L^low^CD69^high^), mature (CD62L^high^CD69^low^), and CD62L^high^CD69^high^ cells of the CD4^+^ SP subset in *S1pr1*^f/f^ and *Lyve1-CRE/S1pr1*^f/f^ thymuses. The mean fluorescence intensity was normalized against CD62L^low^CD69^high^ cells. 6C10 (*n* = 3), HAS (*n* = 3), and S1PR1 (*n* = 3). Data are representative of two independent experiments. These data were evaluated by Student’s *t*-test and represent the mean ± SD (*P < 0.05, **P < 0.005, and ***P < 0.0005). **(C)** *S1pr1* expression in CD4^+^ CD62L^+^ thymocytes of *S1pr1*^f/f^ (*n* = 3) and *Lyve1-CRE/S1pr1*^f/f^ (*n* = 3) thymuses. The expression intensity was normalized with the expression level of *Actb*. Data are representative of two independent experiments. These data were evaluated by Student’s *t*-test and represent the mean ± SD (***P < 0.0005).

We next sought to determine why S1PR1 ablation in *Lyve1*-expressing cells resulted in the accumulation of semi-mature CD4^+^ and CD8^+^ SP T cell subsets in the thymic medulla. As shown in Figures 3D and 4A, further analysis indicated that these CD62L^high^CD69^high^ cells were phenotypically very similar to the egress-competent cells of the thymus in that they were Qa-2^high^6C10^−^ HSA^low^ (33, 34); however, the S1PR1 expression was uniformly low or absent in these cells (Figure 4B). Quantitative PCR analysis confirmed that *S1pr1* expression was strongly attenuated in mature SP thymocytes in *Lyve1-CRE/S1pr1*^f/f^ mice (Figure 4C). This was an unexpected finding, because (1) SP thymocytes most closely resembling recent thymic emigrants (HSA^low^CD62L^high^Qa-2^high^) normally express very high levels of S1PR1 (9, 35) and (2) *S1PR1* was deleted selectively in the cells that expressed the lymphatic endothelial cell-specific marker, Lyve1, in these mice.

### A Large Proportion of Thymocytes, Including Those in the Medulla, Belong to the Lyve1 Lineage

**Figure 5:**
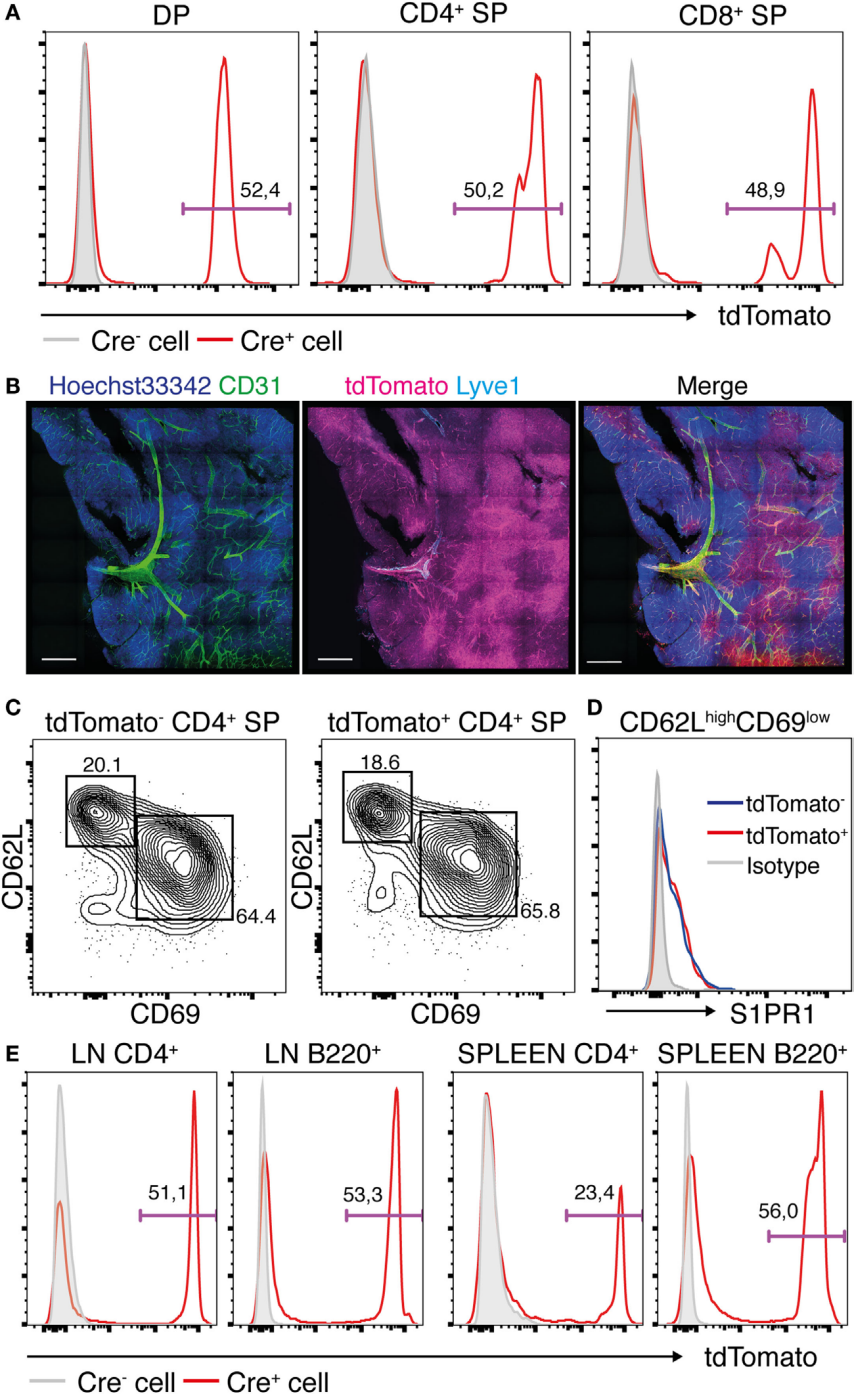
**Most of thymic stromal cells and thymocytes belongs to the Lyve1 lineage**. **(A)** tdTomato expression in the thymocytes of *R26*-tdTomato (Cre^−^) or *Lyve1-CRE/R26*-tdTomato (Cre^+^) mice. **(B)** Histological sections of the thymus from *Lyve1-CRE/R26*-tdTomato reporter mice stained with anti-CD31 (green) and anti-Lyve1 (cyan) antibodies, and the Hoechst 33342 nuclear stain (blue). Cre recombinase-mediated tdTomato expression is shown in magenta. Bars: 500 μm. **(C,D)** Expression of CD62L, CD69, and S1PR1 in tdTomato^−^ or tdTomato^+^ CD4^+^ SP cells of *Lyve1-CRE/R26*-tdTomato mice. S1PR1 expression was examined on mature CD4^+^ SP cells. **(E)** tdTomato expression in the T and B cells in the LNs and spleens of *R26*-tdTomato (Cre^−^) and *Lyve1-CRE/R26*-tdTomato (Cre^+^) mice. *n* = 3 **(A,E)** and *n* = 2 **(B–D)** per group.

The lack of S1PR1 expression in the SP T cell subsets in the *Lyve1-CRE/S1pr1*^f/f^ mice suggested that these cells may belong to the Lyve1 cell lineage, consistent with a recent lineage tracing study, indicating that a small subset of hematopoietic stem cells arise from the Lyve1 lineage cells (23). To determine if the SP T cells belong to the Lyve1 cell lineage, we crossed *Lyve1-CRE* mice with the *Rosa26-*floxed stop*-*tdTomato (*R26*-tdTomato) mice to generate *Lyve1-CRE* reporter mice. In these mice, the *Lyve1* promoter-induced expression of Cre recombinase in Lyve1-expressing cells results in excision of a floxed stop cassette preceding the tdTomato sequence at the *Rosa26* locus, leading to intracellular tdTomato fluorescence. As shown in Figure 5A, flow cytometric analysis of thymocytes from these mice showed strong tdTomato expression in approximately half of the CD4^+^CD8^+^ DP cells and the CD4^+^ and CD8^+^ SP T cells, suggesting that a considerable proportion of these cells are derived from the Lyve1 lineage. Consistent with this possibility, histochemical analysis of thymus revealed that tdTomato was expressed in both the thymic cortex and the medulla, with the medulla exhibiting much stronger fluorescence than the cortex (Figure 5B). Both tdTomato-marked and -unmarked mature thymocytes exhibited comparable expression level of CD62L, CD69, and S1PR1 (Figures 5C,D). The peripheral LNs and spleen also contained large numbers of tdTomato-marked and -unmarked T and B cells (Figure 5E), indicating that Lyve1 and non-Lyve1 lineage cells emigrate from the primary lymphoid tissues to repopulate the peripheral lymphoid tissues. Notably, however, we were unable to detect Lyve1 expression on the cell surface of the tdTomato-marked cells either immunohistochemically (Figure 5B) or by flow cytometry (data not shown). These results collectively indicate that Lyve1 is expressed in a large proportion of lymphocytes during ontogeny and that, in the case of the T cell lineage, both Lyve1 and non-Lyve1 lineage lymphocytes seed and emigrate from the thymus to replenish the recirculating T cell pool in the periphery.

### The SP Subsets in the *Lyve1-CRE/S1pr1*^f/f^ Thymus Are Egress Incompetent

**Figure 6:**
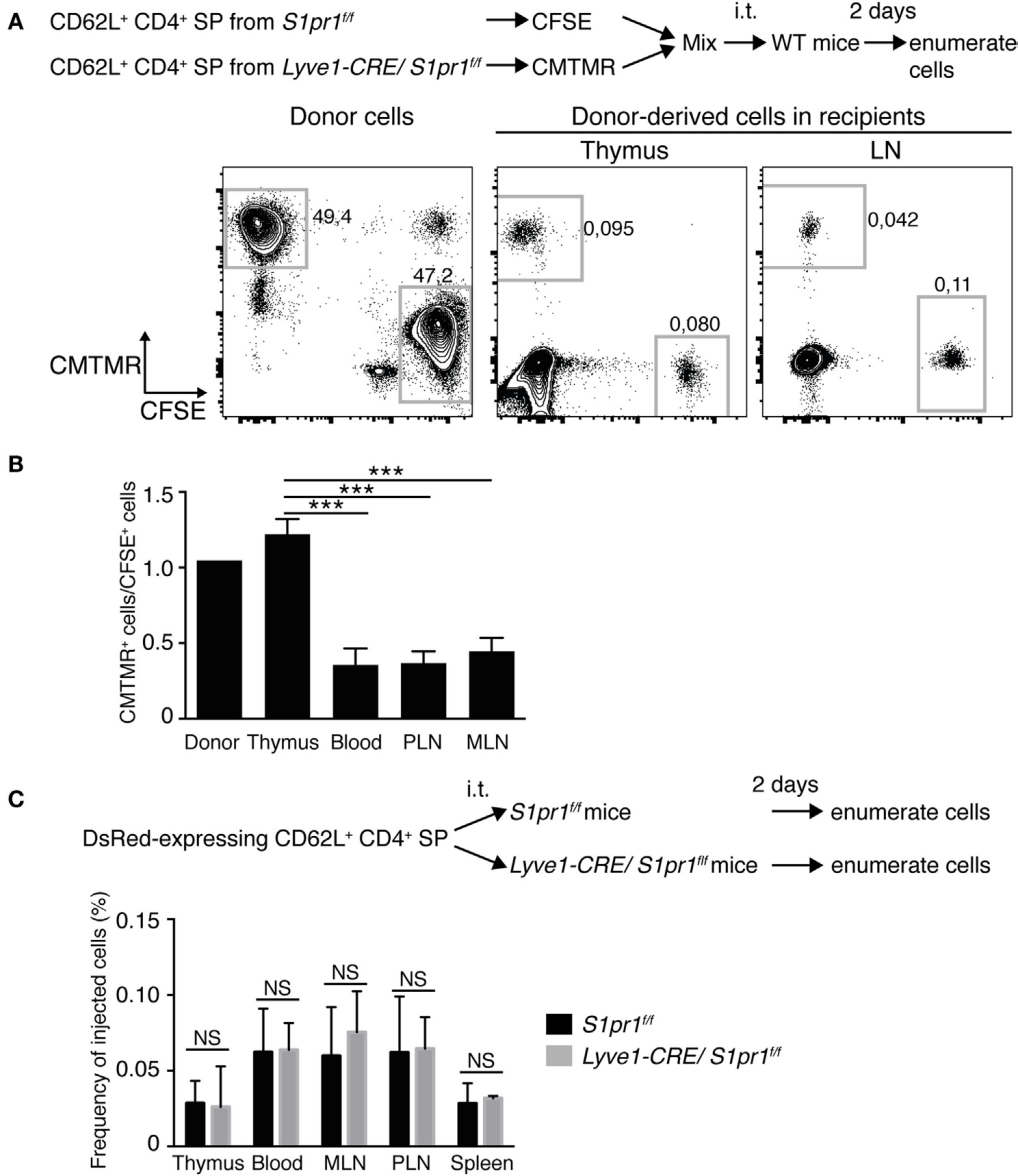
**CD4^+^ SP cells in the *Lyve1-CRE/S1pr1*^f/f^ thymus are egress-incompetent**. **(A)** Egress of *S1pr1*^f/f^ or *Lyve1-CRE/S1pr1*^f/f^ thymocytes from the WT thymus. CD62L^+^ CD4^+^ SP cells of the *S1pr1*^f/f^ or *Lyve1-CRE/S1pr1*^f/f^ thymus were labeled with CFSE or CMTMR, respectively, and cotransferred into the WT thymus in equal numbers (1 × 10^6^ cells per mouse) by intrathymic (i.t.) injection. Two days after the injection, the numbers of donor-derived cells in the thymus, blood, and LNs of recipient mice were analyzed by flow cytometry. **(B)** Ratio of *Lyve1-CRE/S1pr1*^f/f^ CD4^+^ SP cells (CMTMR) to *S1pr1*^f/f^ CD4^+^ SP cells (CFSE) that migrated into the WT recipient’s LNs. PLN, peripheral LNs; MLN, mesenteric LNs. Data are representative of two independent experiments with three mice per group. **(C)** Egress of WT thymocytes from the *S1pr1*^f/f^ or *Lyve1-CRE/S1pr1*^f/f^ thymus. CD62L^+^CD4^+^ SP cells from beta-actin-DsRed mice were injected into the *S1pr1*^f/f^ or *Lyve1-CRE/S1pr1*^f/f^ thymus. Two days later, the frequencies of DsRed^+^ cells in the thymus, blood, and LNs were analyzed by flow cytometry. Data are pooled from two independent experiments (*n* = 3 per group) and represent the mean ± SD. Differences between the groups were evaluated by one-way ANOVA **(B)** or Student’s *t*-test **(C)** (***P < 0.005).

To determine whether the increased SP T cell subsets in the *Lyve1-CRE/S1pr1*^f/f^ thymus were egress defective, we performed intrathymic adoptive transfer assays (36, 37) using fluorescently labeled CD62L^+^CD4^+^ SP thymocytes from *Lyve1-CRE/S1pr1*^f/f^ and *S1pr1*^f/f^ mice. As shown in Figure 6, when a 1:1 mixture of *S1pr1*^f/f^ CD62L^+^CD4^+^ SP T cells and *Lyve1-CRE/S1pr1*^f/f^ CD62L^+^CD4^+^ SP T cells labeled with CFSE and CMTMR, respectively, were cotransferred into the thymus of wild-type (WT) recipients, the *Lyve1-CRE/S1pr1*^f/f^ CD4^+^ SP T cells were retained in the thymus more abundantly than the *S1pr1*^f/f^ CD4^+^ SP T cells, and they migrated much less readily to the recipient’s LNs than did the *S1pr1*^f/f^ CD4^+^ SP T cells (Figures 6A,B). Similar results were obtained when the fluorescent labels were swapped (data not shown), indicating that the fluorescent labels had no effect on lymphocyte migration. In contrast, when DsRed-expressing WT CD62L^+^CD4^+^ SP T cells were adoptively transferred into the thymus of *Lyve1-CRE/S1pr1*^f/f^ or *S1pr1*^f/f^ mice, their migration to the LNs of the two different type of mice was comparable (Figure 6C), indicating that S1PR1 expression of the thymic blood or lymphatic vasculature is dispensable for thymocyte egress. These results collectively demonstrated that *Lyve1-CRE/S1pr1*^f/f^ CD4^+^ SP T cells exhibit defective thymus egress and that the T cell intrinsic, but not stromal cell-associated S1PR1 plays a critical role in T cell emigration from the thymus. Our results also suggested that subsets of both T and B cells arise from the Lyve1 linage.

## Discussion

The thymus plays an integral role in the production, maturation, and export of T cells to the periphery. Previous studies demonstrated that the thymocyte egress occurs *via* two different routes, blood vessels (10, 12) and lymphatics (12–14), and that thymocyte egress *via* blood vessels is critically regulated by lymphocyte-intrinsic S1PR1 and pericyte-derived S1P (10). However, S1PR1 is also abundantly expressed by lymphatic endothelial cells (38) and appears to be activated constitutively in lymphatics (11), although S1PR1’s role in lymphatic endothelial cells remains unresolved. In the present study, we sought to address the S1PR1’s role in lymphatic endothelial cells by deleting the *S1pr1* gene in Lyve1-expressing cells by generating *Lyve1-CRE/S1pr1*^f/f^ mice. Lyve1 is expressed in a majority of lymphatics at the protein level and, hence, has been used as a lymphatic endothelial cell marker in a number of previous studies (16, 39–43), although its expression in other cell types has been reported (19, 21–23).

Our study showed that the *Lyve1-CRE/S1pr1*^f/f^ mice exhibited marked increases in thymic CD4^+^ and CD8^+^ SP T cell subsets and enlarged thymic medullas compared with those of control *S1pr1*^f/f^ mice or WT mice. The SP T cells from the mutant mice exhibited a mature T cell phenotype (6C10^−^ HSA^low^ Qa-2^+^ CD62L^+^), superficially resembling T cells leaving the thymus (33); however, they lacked S1PR1 expression, which was previously reported to be essential for thymocyte egress (9). The T cells retained in the *Lyve1-CRE/S1pr1*^f/f^ thymus also exhibited much higher CD69 expression than egress-competent cells (34), which was probably due to the lack of S1PR1 expression, since S1PR1 signaling is critical for suppressing CD69 surface expression (9, 44) (Figure S2 in Supplementary Material). The absence or paucity of S1PR1 expression in these cells was surprising, since we used the *Lyve1-CRE* system to conditionally disrupt *S1pr1* in lymphatic endothelial cells. We therefore sought to track Lyve1 lineage cells and their descendants by generating the *Lyve1-CRE/R26*-tdTomato mice, in which a red fluorescent protein, tdTomato, is expressed in Lyve1-expressing cells and the cells in which Lyve1 has been expressed. When isolated cells from the peripheral lymphoid tissues of these mice were examined by flow cytometry, reporter gene activation was observed in substantial proportions of T and B lymphocytes, even though they were completely devoid of Lyve1 expression on the cell surface. Furthermore, histochemical analysis confirmed the abundance of tdTomato-marked cells in the thymus of these mice, particularly in the medullary compartments where egress-competent cells are thought to mainly reside (33, 34, 45). A majority of the tdTomato-marked and -unmarked mature thymocytes expressed S1PR1. Collectively, these results strongly suggest that the CD4^+^ and CD8^+^ SP T cells accumulating in the *Lyve1-CRE/S1pr1*^f/f^ mice are Lyve1 lineage descendants that are devoid of S1PR1 and therefore unable to undergo thymus egress.

Upon surveying the literature, we found reports describing the Lyve1 expression in non-lymphatic endothelial cell lineages during ontogeny (21, 23, 46). Of note is a recent study by Lee (23) showing that *Lyve*1 expression first appears in the mouse yolk sac at E8.5 and that Lyve1 is detected on the cell surface of ~50% of the c-kit^+^ hematopoietic cell precursors and most of the CD31^+^ hemogenic endothelial cells in the yolk sac by E10.5 (23). *Lyve1-CRE* lineage tracing using *Lyve1-CRE/R26-*YFP mice showed that at least 30% of the hematopoietic precursors in the fetal liver and adult bone marrow express the reporter gene, indicating that they are derived from the Lyve1 lineage, although Lyve1 protein was not detected on the cell surface of bone marrow cells in adult mice. Collectively, these data indicate that *Lyve1* expression is developmentally regulated in hematopoietic cells and that hematopoietic precursor cells arise from Lyve1 and non-Lyve1 lineages in mice. Our results are consistent with the Lee’s finding (23) and extend them by showing that a significant proportion of thymocytes and peripheral T cells are derived from the Lyve1 cell lineage and that thymocytes of this lineage in *Lyve1-CRE/S1pr1*^f/f^ mice fail to leave the thymus due to the absence of S1PR1 expression. Thus, our study verifies that cell exit from the thymus is mediated by thymocytes’ S1PR1 expression by using a completely different model system from that described in previous studies (9, 10). Although our study do not support the involvement of S1PR1 on thymic stromal cells in the regulation of thymocyte egress, future work involving the use of conditional knockout mice in which *S1pr1* is postnatally deleted in the thymic stromal cells should clarify this issue.

The biological significance of the *Lyve1*^+^/*Lyve*1^−^ T cell lineages is currently unclear. Flow cytometric analysis of the two populations showed that their phenotypes were comparable, and histochemical analysis indicated that they were randomly distributed in peripheral lymphoid tissues (data not shown). The finding that T and B cells in the LNs and spleen consisted of tdTomato-marked and -unmarked populations suggests that lymphocytes descending from both Lyve1 and non-Lyve1 lineages have the ability to emigrate from the respective primary lymphoid tissues to repopulate the peripheral lymphoid tissues. Further investigation is required to understand the biological significance of *Lyve1* expression in the lymphocyte lineage.

## Author Contributions

AT conceived and designed the study; acquired, analyzed, and interpreted the data; and drafted the article; MH acquired, analyzed, and interpreted the data; PR, NS, MS, and SJ analyzed and interpreted the data; SS contributed unpublished essential data; MM conceived and designed the study; analyzed and interpreted the data; and drafted the article.

## Conflict of Interest Statement

The authors declare that the research was conducted in the absence of any commercial or financial relationships that could be construed as a potential conflict of interest.
