# Supplementary material for: Thymocytes in Lyve1-CRE/S1pr1f/f Mice Accumulate in the Thymus due to Cell-Intrinsic Loss of Sphingosine-1-Phosphate Receptor Expression
Source: Front Immunol. 2016 Nov 8;7:489. doi: 10.3389/fimmu.2016.00489 (PMC5099144; doi:10.3389/fimmu.2016.00489)
Supplement: Supplementary file 1 [file Presentation_1.PPTX]

## Slide 1
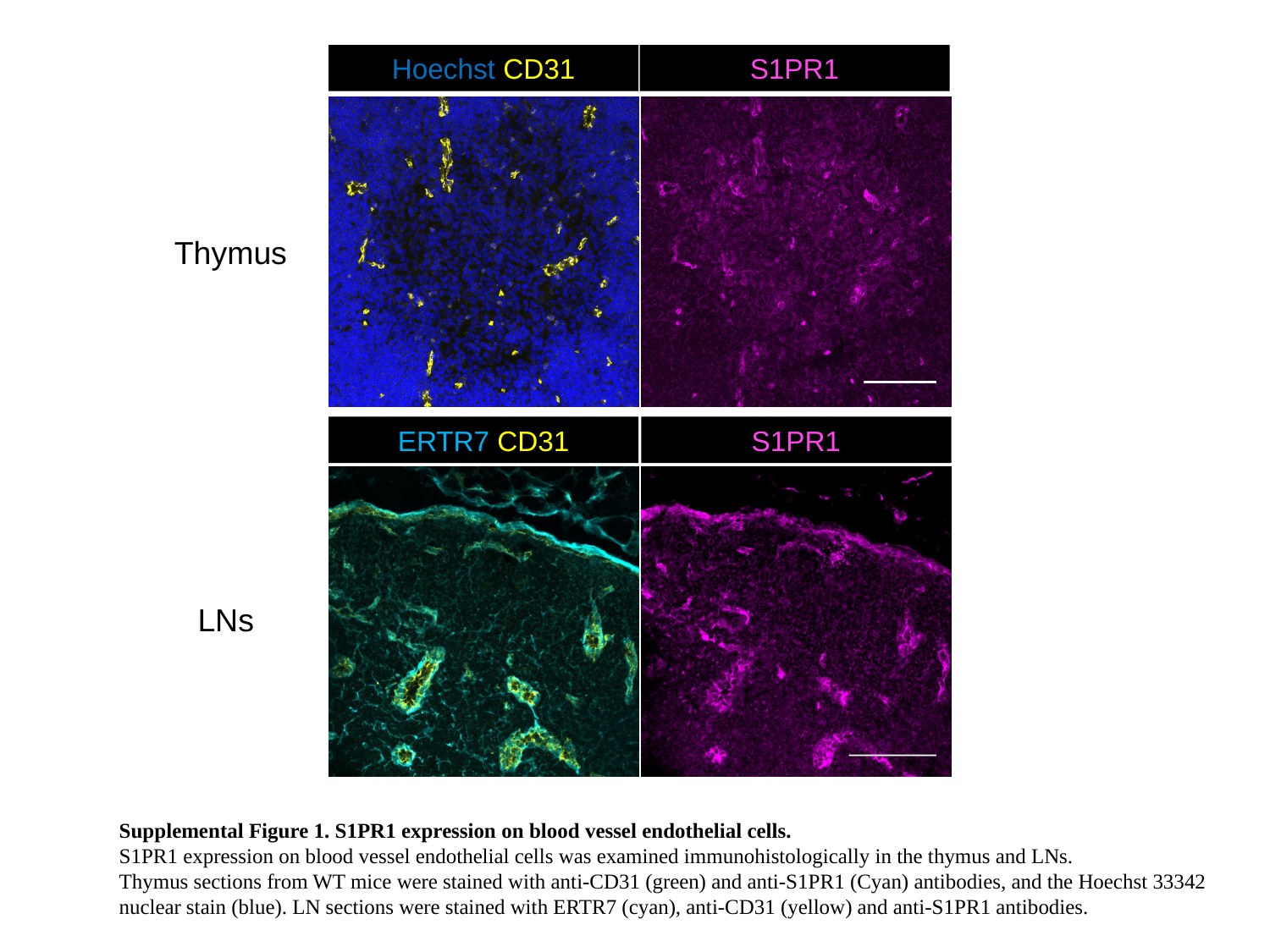

Hoechst CD31
S1PR1
Thymus
ERTR7 CD31
S1PR1
LNs
Supplemental Figure 1. S1PR1 expression on blood vessel endothelial cells.
S1PR1 expression on blood vessel endothelial cells was examined immunohistologically in the thymus and LNs.
Thymus sections from WT mice were stained with anti-CD31 (green) and anti-S1PR1 (Cyan) antibodies, and the Hoechst 33342
nuclear stain (blue). LN sections were stained with ERTR7 (cyan), anti-CD31 (yellow) and anti-S1PR1 antibodies.
