# Supplementary material for: Thymocytes in Lyve1-CRE/S1pr1f/f Mice Accumulate in the Thymus due to Cell-Intrinsic Loss of Sphingosine-1-Phosphate Receptor Expression
Source: Front Immunol. 2016 Nov 8;7:489. doi: 10.3389/fimmu.2016.00489 (PMC5099144; doi:10.3389/fimmu.2016.00489)
Supplement: Supplementary file 2 [file Presentation_2.PPTX]

## Slide 1
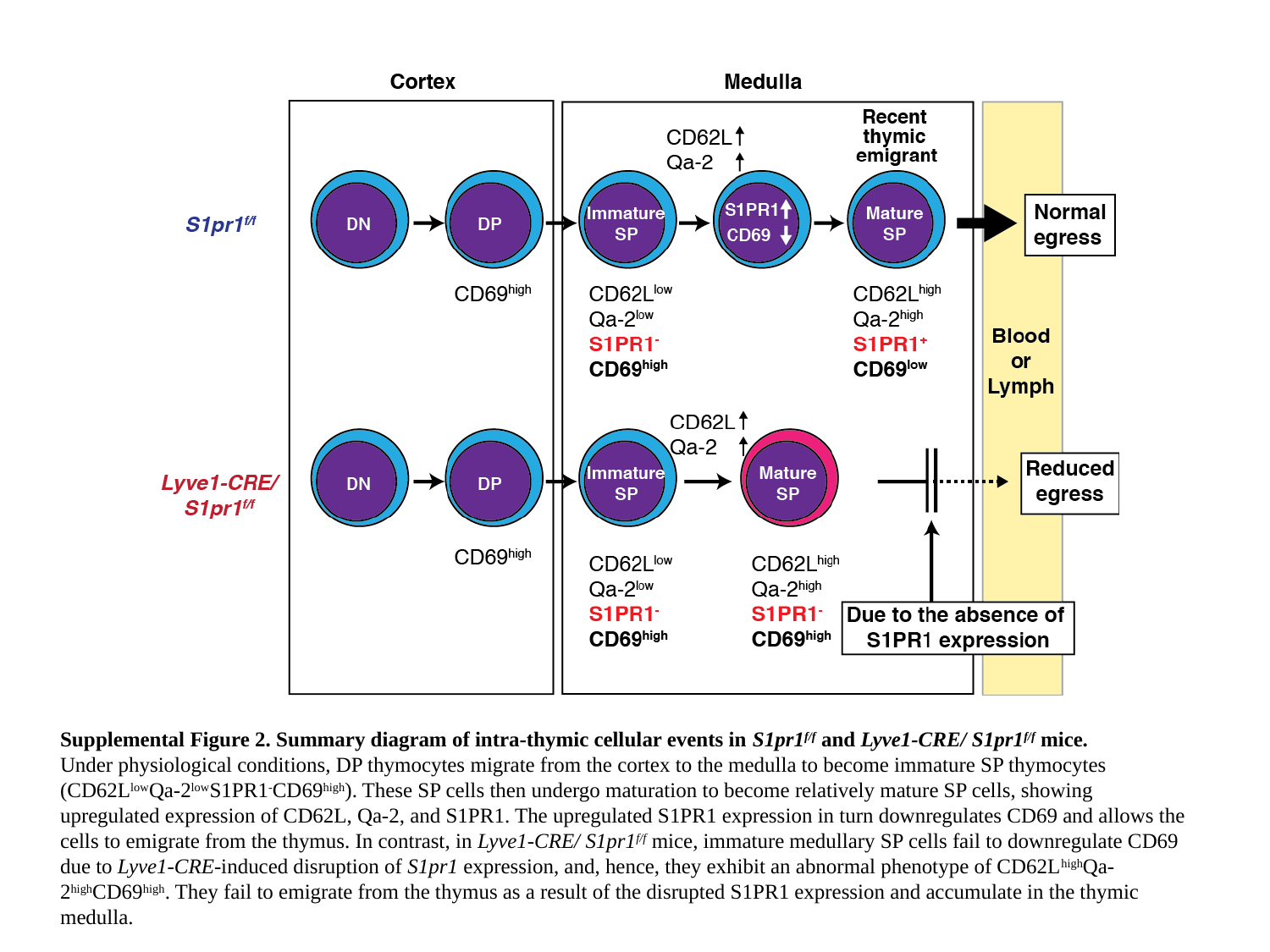

Supplemental Figure 2. Summary diagram of intra-thymic cellular events in S1pr1f/f and Lyve1-CRE/ S1pr1f/f mice.
Under physiological conditions, DP thymocytes migrate from the cortex to the medulla to become immature SP thymocytes
(CD62LlowQa-2lowS1PR1-CD69high). These SP cells then undergo maturation to become relatively mature SP cells, showing upregulated expression of CD62L, Qa-2, and S1PR1. The upregulated S1PR1 expression in turn downregulates CD69 and allows the cells to emigrate from the thymus. In contrast, in Lyve1-CRE/ S1pr1f/f mice, immature medullary SP cells fail to downregulate CD69 due to Lyve1-CRE-induced disruption of S1pr1 expression, and, hence, they exhibit an abnormal phenotype of CD62LhighQa-2highCD69high. They fail to emigrate from the thymus as a result of the disrupted S1PR1 expression and accumulate in the thymic medulla.
